# Anti-PD-1 Autoantibody Predicts Survival of Patients With Hepatocellular Carcinoma Receiving Atezolizumab/Bevacizumab

**DOI:** 10.1016/j.gastha.2024.07.018

**Published:** 2024-08-02

**Authors:** Yuki Sasaki, Kazuyuki Matsumoto, Akinobu Takaki, Takuya Adachi, Masahiro Takahara, Keita Ozato, Yasuto Takeuchi, Masahiko Sue, Nozomi Miyake, Nozomu Wada, Hideki Onishi, Hidenori Shiraha, Takashi Oda, Koichiro Tsutsumi, Kazuhiro Nouso, Kazuya Kariyama, Hiroaki Hagihara, Akio Moriya, Motoyuki Otsuka

**Affiliations:** 1 Department of Gastroenterology and Hepatology, Okayama University Graduate School of Medicine, Dentistry and Pharmaceutical Sciences, Okayama, Japan; 2 Department of Gastroenterology, Okayama City Hospital, Okayama, Japan; 3 Department of Gastroenterology, Sumitomo Besshi Hospital, Niihama, Ehime, Japan; 4 Department of Gastroenterology, Mitoyo General Hospital, Kanonji, Kagawa, Japan

**Keywords:** Immune Response, Programmed Cell Death-1, Biomarker, Immune Checkpoint Inhibitors, Overall Survival

## Abstract

**Background and Aims:**

Methods for predicting therapeutic response to immune checkpoint inhibitors in cancer therapy are in high demand. In patients with advanced hepatocellular carcinoma (HCC), atezolizumab (anti-programmed cell death-ligand 1 [PD-L1]) and bevacizumab (anti-vascular endothelial growth factor) combination therapy (Atezo/Bev therapy) is a first-line treatment. However, no reliable biomarkers are currently available to predict its efficacy. Here, we examined serum anti-PD-1 autoantibody levels as candidate biomarkers.

**Methods:**

We prospectively enrolled 63 patients with advanced HCC who received Atezo/Bev therapy. Serum anti-PD-1 autoantibody levels were measured before treatment using an indirect enzyme-linked immunosorbent assay. The correlation between the titers and response to therapy was statistically examined.

**Results:**

Serum anti-PD-1 autoantibody levels were not significantly associated with the treatment response in any patient. However, when examining only patients who received the Atezo/Bev as their first-line therapy, higher anti-PD-1 autoantibody levels were significantly associated with worse overall survival rates. The titer was an independent risk factor for poor prognosis (odds ratio [OR] = 7.8, *P* = .013), in addition to a higher neutrophil-to-lymphocyte ratio (OR = 7.1, *P* = .009) and lower albumin levels (OR = 14.2, *P* = .003).

**Conclusion:**

Serum anti-PD-1 autoantibody levels correlated with the overall survival rate in patients who received Atezo/Bev as first-line therapy. Serum anti-PD-1 autoantibody levels may serve as new biomarkers for predicting the efficacy of immune checkpoint inhibitors in patients with HCC.

## Introduction

Hepatocellular carcinoma (HCC) is the most common primary malignancy of the liver and is the fourth leading cause of cancer-related death worldwide.^1^ For advanced HCC, systemic treatment is selected according to tumor burden, liver reservoir function, and patient performance status.^2^ According to the clinical guideline on systemic therapy for advanced HCC, the combination of immune checkpoint inhibitor (ICI) anti-programmed cell death-ligand-1 (PD-L1) antibody atezolizumab and anti-vascular endothelial growth factor antibody bevacizumab (Atezo/Bev) is currently recommended as a first-line treatment in patients with preserved liver function,^3^ as well as the ICI combination treatment that contains anti-CTLA-4 antibody tremelimumab and anti-PD-L1 antibody durvalmab (Single Tremelimumab Regular Interval Durvalmab regimen).^4^, ^5^, ^6^

Although Atezo/Bev combination therapy shows a strong effect, nearly 20% of treated patients show progressive disease (PD).^7^^,^^8^ Because the multikinase inhibitors lenvatinib and sorafenib are also considered first-line therapies, predicting the effects of Atezo/Bev is important for determining an appropriate regimen.

Among the predictors, tumor tissue-based biomarkers, such as PD-L1 expression levels in tumor tissues, remain the most useful immune-based treatment effect biomarkers in current clinical practice.^7^^,^^9^^,^^10^ However, PD-L1 expression is highly heterogeneous and may be altered by exposure to prior therapy.^11^^,^^12^ In addition, patients with advanced HCC who are eligible for immunotherapy are often diagnosed without a tumor biopsy. Therefore, it is important to develop noninvasive blood-based biomarkers to predict the effectiveness of ICI treatment.

Among the possible noninvasive markers, the peripheral blood neutrophil-to-lymphocyte ratio (NLR) is a prognostic blood-based marker for the treatment of HCC including Atezo/Bev therapy.^13^^,^^14^ Some cytokine levels, such as CXCL9 or IL-6, have also been associated with the response to Atezo/Bev therapy. However, these have not been sufficiently reliable or have not yet been confirmed by other studies.^15^^,^^16^

Autoantibodies (AAbs) may emerge in the patients with several cancers.^17^ Anti-PD-1 AAb has been detected in the sera of patients with several cancer types.^18^ However, its prognostic utility has not yet been reported, except for non-small cell lung cancer, with the very limited number of cases examined.^18^ Anti-PD-1 AAb may affect the therapeutic effectiveness of the anti-PD-L1 antibody externally administered as a therapeutic option, such as atezolizumab, because it has the same target pathway. We hypothesized that, if anti-PD-1 AAb exists in the sera of the cases with advanced HCC, it will affect the efficacy of the Atezo/Bev therapy. Therefore, in this study, we measured serum anti-PD-1 AAb levels in patients with HCC who underwent Atezo/Bev therapy and determined whether the serum anti-PD-1 AAb levels can be used as prognostic biomarkers for patients with HCC who have received Atezo/Bev therapy.

## Methods

### Patients and Study Design

Patients with advanced HCC from 4 institutions participating in the Okayama Liver Cancer Group were prospectively registered and underwent Atezo/Bev therapy. This study defined advanced HCC in patients who were not eligible for curative or local therapies such as radiofrequency ablation (RFA) or resection and patients with metastatic disease. HCC was diagnosed with dynamic contrast-enhanced computed tomography (CT) or dynamic contrast-enhanced magnetic resonance imaging (MRI).^2^ A total of 63 patients were enrolled between November 2020 and October 2022. None of the patients received regimens with anti-PD-1 or anti-PD-L1 antibodies before the Atezo/Bev administration. Patients were treated with Atezo/Bev treatment every 3 weeks, and the therapeutic response was evaluated according to the guidelines of the Response Evaluation Criteria in Solid Tumors (RECIST) using dynamic CT or MRI.^7^^,^^19^ The initial treatment efficacy assessment was performed using dynamic CT or MRI approximately 6 weeks after Atezo/Bev introduction whenever possible, according to the phase III IMbrave150 study, followed by another dynamic CT or MRI every 9–12 weeks as required depending on the patient's condition. In some cases, additional dynamic CT or MRI examinations were performed even before 9–12 weeks. The number of subgroup patients who received Atezo/Bev as the first-line treatment was 43. Data on the clinical and biochemical characteristics of the patients were collected at treatment initiation. We collected follow-up serum from the first-line therapy group at the first evaluation for 4 patients with complete response (CR) and 5 patients with PD to determine anti-PD-1 AAb changes. The overall response rate (ORR) was defined as the proportion of patients who achieved a CR or partial response (PR), and disease control rate (DCR) was defined as the proportion of patients who achieved CR, PR, or stable disease (SD) as their best overall response according to the RECIST criteria. Written informed consent was obtained from each patient before study enrollment. The study protocol conformed to the ethical guidelines of the Declaration of Helsinki. The study was approved by the institutional ethics review committee of Okayama University (KEN1709-023).

### Blood Sample Collection and Preparation

Fasting blood samples were collected from all patients. Sera were collected at the time of admission for Atezo/Bev treatment. Aliquots were stored at −30 °C until further analysis.

### Measurement of the Anti-PD-1 Autoantibody Levels

To measure the anti-PD-1 autoantibody levels, an indirect enzyme-linked immunosorbent assay (ELISA) was performed according to the method used in our previous report, with some modifications.^20^ Briefly, plate and buffer system for ELISA (Ab-Match Universal Kit 5310) was purchased from MBL (Nagano, Japan). Ninety-six well flat bottom plates for ELISA included in the kit were coated with 100 μL of 1 μg/mL recombinant PD-1 (Abnova, Taipei, Taiwan) at 4 °C overnight. The next day, the plates were washed twice with wash buffer and blocked with blocking buffer. After incubation for 1 hour at 37 °C, the sera were added to the wells after the sample dilution at 1:50 with sample diluent buffer. The positive and negative controls were defined based on our previous results. The positive and negative controls were the sera of 63 healthy volunteers with the highest and lowest titers of anti-PD-1 autoantibodies, respectively. They were also added to the assay to confirm the success. After incubation for 1 hour at room temperature, the wells were washed 4 times, and 100 μL of 1:100 diluted peroxidase-labeled anti-human IgG (LGC Clinical Diagnostics, Inc, Milford, MA) were applied to the wells. The plates were then incubated for 1 hour at room temperature. After incubation, the plates were washed 4 times, and the substrate solution was added. The optical density at 450 nm was measured using a Model 680 microplate reader (Spectrophotometer Type 1510) (ThermoFisher Scientific, Vantaa, Finland). The relative ratio of the data from the samples to the data from negative controls was defined as the relative ratio of anti-PD-1 autoantibody. The threshold of the anti-PD-1 autoantibody levels for grouping cases with lower and higher titers of anti-PD-1 autoantibody in this study was set as the median value of all cases tested. The measurements were duplicated for each sample, and the average values were adopted. The median value of positive control was 1.897.

### Statistical Analysis

Patients were divided into 2 groups (high anti-PD-1 AAb vs low anti-PD-1 AAb levels). One-way analysis of variance followed by the Kruskal-Wallis test was used to assess nonparametric data between the 2 groups. Pearson’s product-moment correlation coefficient was used to assess correlations and analyze categorical data between the 2 groups. In the survival analysis, the end point of overall survival (OS) was defined as the time of death from the date of treatment initiation. The end point of progression-free was determined as the time of disease progression assessed by RECIST (version 1.1) or death, whichever occurred first, after the day of treatment initiation. Differences in OS and progression-free survival (PFS) were analyzed using the Kaplan-Meier method and Wilcoxon test. Factors associated with improved OS were analyzed using univariate and multivariate Cox proportional hazards regression models. For the dichotomizing factors, we used the median value as the cut-off.

For the follow-up analyses, anti-PD1-AAb titers at the pretreatment and first evaluation points were compared in 4 CR and 5 PD patients using the Wilcoxon signed rank test.

A *P* value < .05 was considered statistically significant. JMP software (version 13; SAS, Cary, NC) was used for all analyses.

## Results

### Participants’ Characteristics and the Therapeutic Outcomes by Atezo/Bev Therapy

The baseline clinical and laboratory characteristics of all the participants are shown in Table 1. The median age of the participants was 73 years, and 76.1% were male. Alcohol-related cirrhosis (30.2%) was the most common etiology of HCC. Most participants were classified as Child-Pugh class A (96.8%). The Barcelona Clinic Liver Cancer stages were determined just before Atezo/Bev’s introduction. Of the 36 patients who received local therapy before Atezo/Bev administration, 26 were treated for hepatectomy and 21 for RFA (11 received both treatments). The number of patients who received Atezo/Bev as the first-line chemotherapy was 43 (68.2%). The study showed that the ORR and DCR evaluated by RECIST (version 1.1) were 40.3% and 62.9%, respectively (CR/PR/SD/PD/not evaluable: 3/22/14/23/1) (Figure A1A), and the one-year OS rate was 70.0%, with a median follow-up duration of 12.6 months (Figure A1B). The median PFS rate was 113 days. Twenty-three patients (36.5%) died due to HCC during the observation period.

**Table 1:** Baseline Characteristics of All Patients

Patient characteristics	Value (median, range)
Age, y	73 (46–87)
Sex, male/female	48/15 (76%/24%)
ECOG performance status, 0/1/2/3/4	58/4/1/0/0 (92%/6%/1%/0%/0%)
Etiology of HCC	
Hepatitis B	12 (19%)
Hepatitis C	18 (29%)
Alcohol	19 (30%)
NASH	9 (14%)
Others or unknown	5 (8%)
Child-Pugh score, 5/6/7	32/29/2 (51%/46%/3%)
PLT, 10^4^/μL	16.4 (6–57.2)
NLR	2.57 (0.74–23)
T-BiL, mg/dL	0.74 (0.27–2.36)
Albumin, g/dL	3.7 (2.8–4.7)
Cr, mg/dL	0.79 (0.45–1.54)
Ferritin, ng/mL	169 (20–1941)
PT-INR	1.04 (0.91–1.29)
AFP, ng/mL	141 (1.3–194,305)
DCP, mAU/mL	341 (10–332,649)
Distant metastasis, present/absent	27/36 (43%/57%)
Vascular invasion, present/absent	23/40 (37%/63%)
BCLC stage, A/B/C	3/17/43 (5%/27%/68%)
Prior local therapy, present/absent	36/27 (57%/43%)
Treatment line, 1st/2nd/3rd/4th	43/13/2/5 (68%/21%/3%/8%)
Observation time, d	378 (26–875)

AFP, alfa-fetoprotein; BCLC, Barcelona Clinic Liver Cance; Cr, creatinine; DCP, des-γ-carboxy prothrombinr; ECOG, Eastern Cooperative Oncology Group; HCC, hepatocellular carcinoma; NASH, nonalcoholic steatohepatitis; NLR, neutrophil lymphocyte ratio; PLT, platelets; PT-INR, prothrombin time-international normalized ratio; T-Bil, total bilirubin.

Similar analyses were conducted for a limited number of patients who received Atezo/Bev as their first-line therapy (Table 2). The median age, sex, baseline liver disease, and liver function of this subgroup were similar to those of all patients. The ORR and DCR were 52.4% and 76.2%, respectively (CR/PR/SD/PD/not evaluable: 2/20/10/10/1) (Figure A1C), and the one-year OS rate was 79.1%, with a median follow-up duration of 13.4 months (Figure A1D). The median PFS was 126 days. Eleven patients (25.5%) died due to HCC during the observation period among the subgroup patients.

**Table 2:** Baseline Characteristics of the Patients Who Used Atezo/Bev as Their First-Line Therapy

Patient characteristics	Value (median, range)
Age, y	73 (46–87)
Sex, male/female	31/12 (72%/28%)
ECOG performance status, 0/1/2/3/4	40/3/0/0/0 (93%/7%/0%/0%/0%)
Etiology of HCC	
Hepatitis B	6 (14%)
Hepatitis C	12 (28%)
Alcohol	13 (30%)
NASH	7 (16%)
Others or unknown	5 (12%)
Child-Pugh score, 5/6/7	28/13/2 (65%/30%/5%)
PLT, 10^4^/μL	16.8 (6–39)
NLR	2.7 (0.74–23)
T-BiL, mg/dL	0.79 (0.37–2.36)
Albumin, g/dL	3.8 (2.9–4.7)
Cr, mg/dL	0.74 (0.45–1.29)
Ferritin, ng/mL	169 (20–1941)
PT-INR	1.06 (0.95–1.29)
AFP, ng/mL	114 (1.3–194,305)
DCP, mAU/mL	350 (10–332,649)
Distant metastasis, present/absent	17/26 (40%/60%)
Vascular invasion, present/absent	16/27 (37%/63%)
BCLC stage, A/B/C	2/11/30 (5%/25%/70%)
Prior local therapy, present/absent	20/23 (47%/53%)
Protocols for second line and subsequent therapies	21 (49%)
2nd: Lenvatinib/Cabozantinib/Sorafenib	19/1/1
3rd: Lenvatinib/Cabozantinib/Sorafenib/STRIDE/Atezo/Bev	1/3/2/2/1
4th: Cabozantinib/Sorafenib/Regorafenib/Atezo/Bev	1/1/1/1
Observation time, d	403 (26–840)

AFP, alfa-fetoprotein; Atezo/Bev, atezolizumab and bevacizumab; BCLC, Barcelona Clinic Liver Cancer; Cr, creatinine; DCP, des-γ-carboxy prothrombin; ECOG, Eastern Cooperative Oncology Group; HCC, hepatocellular carcinoma; NASH, nonalcoholic steatohepatitis; NLR, neutrophil lymphocyte ratio; PLT, platelets; PT-INR, prothrombin time-international normalized ratio; STRIDE, Single Tremelimumab Regular Interval Durvalmab; T-Bil, total bilirubin.

### Overall Survival Rate Was Significantly Associated With the Serum Anti-PD-1 Autoantibody Levels Among the Patients Using Atezo/Bev as the First-Line Therapy

Because we hypothesized that serum anti-PD-1 AAb levels may affect the therapeutic effectiveness of Atezo/Bev due to competition against PD-1, we compared the clinical outcomes of patients who received Atezo/Bev treatment according to their serum anti-PD-1 AAb levels. Patients were divided into 2 groups with higher and lower median serum anti-PD-1 AAb levels (median 0.57), with 32 patients in the higher group and 31 in the lower group.

First, we compared the characteristics of the patients with high and low serum anti-PD-1 AAb levels. The group with higher anti-PD-1 AAb levels had a higher male ratio, lower NLR, lower prior local therapy rates, and higher ferritin levels (Table 3). When limited to patients who received Atezo/Bev as the first-line therapy, patients with higher anti-PD-1 AAb levels showed a significantly higher male ratio and higher ferritin levels (Table 4).

**Table 3:** Characteristics of All Patients According to Their Serum Anti-PD-1 Autoantibody Levels

Patient characteristics	Value (median, range)		*P* value
	Anti-PD-1 autoantibody median 0.57 (range; 0.28–3.19)		
	Low (N = 31)	High (N = 32)	
	<0.57	≥0.57	
Age, y	73 (49–87)	73 (46–86)	.928
Sex, male/female	20/11	28/4	.032^a^
ECOG performance status, >0/0	4/27	1/31	.072
Etiology of HCC, NASH/others	5/26	4/28	.680
Child-Pugh score, 5/6/7	16/14/1	16/15/1	.990
PLT, 10^4^/μL	15.9 (6–37)	16.4 (6.9–57.2)	.611
NLR	2.8 (1.06–23)	1.95 (0.74–6.76)	.032^a^
T-BiL, mg/dL	0.7 (0.27–2.13)	0.8 (0.34–2.36)	.332
Albumin, g/dL	3.7 (2.9–4.7)	3.7 (2.8–4.5)	.895
Cr, mg/dL	0.79 (0.45–1.18)	0.77 (0.46–1.54)	.917
Ferritin, ng/mL	103 (40–1941)	250 (20–1286)	.018^a^
PT-INR	1.01 (0.91–1.17)	1.04 (0.93–1.29)	.588
AFP, ng/mL	205 (1.3–122,480)	131 (1.7–194,305)	.491
DCP, mAU/mL	678 (17–202,087)	431 (10–332,649)	.971
Distant metastasis, present/absent	12/19	15/17	.512
Vascular invasion, present/absent	9/22	14/18	.225
BCLC stage, A, B/C	11/20	9/23	.530
Prior local therapy, present/absent	22/9	14/18	.030^a^
Protocols for the second line and subsequent therapies: present/absent	15/16	16/16	.898
Observation time, d	363 (87–875)	428 (26–840)	.907

Data are expressed as median [interquartile range] or number (percentage).

AFP, alfa-fetoprotein; BCLC, Barcelona Clinic Liver Cancer; Cr, creatinine; DCP, des-γ-carboxy prothrombin; ECOG, Eastern Cooperative Oncology Group; HCC, hepatocellular carcinoma; NASH, nonalcoholic steatohepatitis; NLR, neutrophil lymphocyte ratio; PLT, platelets; PT-INR, prothrombin time-international normalized ratio; T-BIL, total bilirubin.

a *P* < .05.

**Table 4:** Characteristics of the Patients Who Received Atezo/Bev as Their First-Line Therapy According to the Serum Anti-PD-1 Autoantibody Levels

Patient characteristics	Value (median, range)		*P* value
	Anti-PD-1 autoantibody median 0.57 (range; 0.28–3.19)		
	Low (N = 20)	High (N = 23)	
	<0.57	≥0.57	
Age, y	73 (49–87)	73 (46–86)	.788
Sex, male/female	11/9	20/3	.019∗
ECOG performance status, >0/0	3/17	0/23	.054
Etiology of HCC, NASH/others	4/14	3/19	.477
Child-Pugh score, 5/6/7	14/5/1	14/8/1	.784
PLT, 10^4^/μL	16.3 (6–37)	16.9 (8.6–39)	.278
NLR	2.8 (1.06–23)	1.96 (0.74–5.11)	.173
T-BiL, mg/dL	0.7 (0.37–2.13)	0.8 (0.37–2.36)	.400
Albumin, g/dL	3.8 (2.9–4.7)	3.8 (2.9–4.5)	.722
Cr, mg/dL	0.67 (0.45–1.17)	0.75 (0.46–1.29)	.279
Ferritin, ng/mL	84 (40–1941)	298 (20–1286)	.036∗
PT-INR	1.06 (0.95–1.17)	1.06 (0.96–1.29)	1.000
AFP, ng/mL	78.2 (1.3–31,410)	125 (1.7–194,305)	.242
DCP, mAU/mL	78 (17–121,757)	878 (10–332,649)	.277
Distant metastasis, present/absent	7/13	10/13	.570
Vascular invasion, present/absent	5/15	11/12	.122
BCLC stage, A, B/C	8/12	5/18	.193
Prior local therapy, present/absent	12/8	8/15	.098
Protocols for the second line and subsequent therapies: present/absent	9/11	12/11	.638
Observation time, d	377 (87–806)	453 (26–840)	.932

Data are expressed as median [interquartile range] or number (percentage).

AFP, alfa-fetoprotein; BCLC, Barcelona Clinic Liver Cancer; Cr, creatinine; DCP, des-γ-carboxy prothrombin; ECOG, Eastern Cooperative Oncology Group; HCC, hepatocellular carcinoma; NASH, nonalcoholic steatohepatitis; NLR, neutrophil lymphocyte ratio; PLT, platelets; PT-INR, prothrombin time-international normalized ratio; T-BIL, total bilirubin.

a *P* < .05.

**Figure 1:**
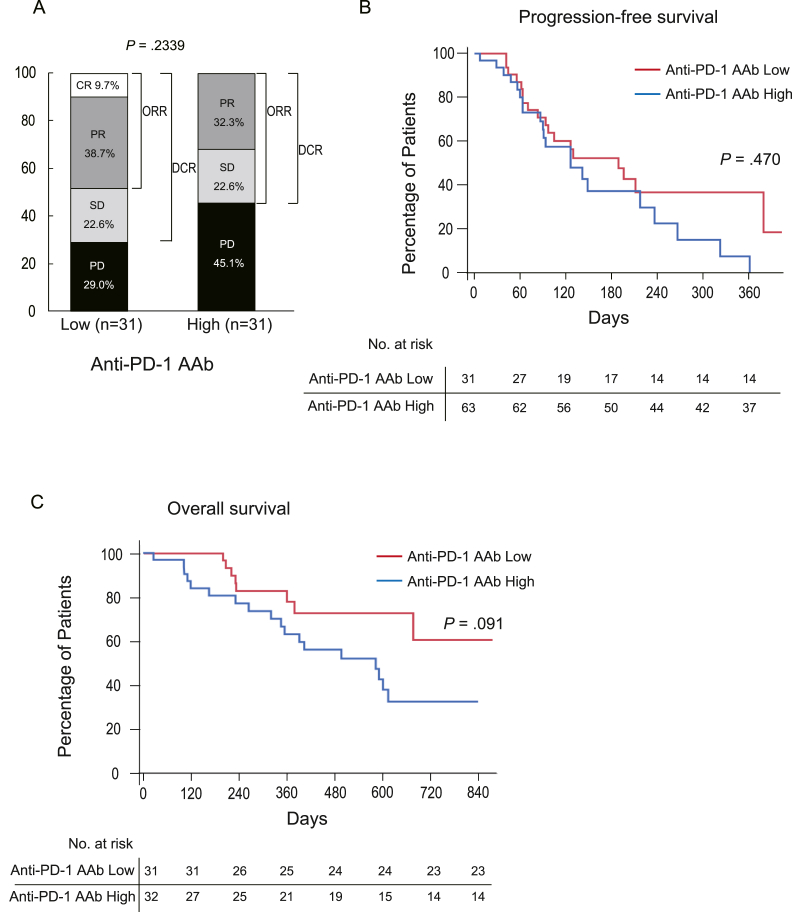
Treatment responses among all patients according to the serum anti-PD-1 autoantibody levels. (A) The bar chart showing the best response to Atezo/Bev therapy determined by the radiological assessment among all patients when the patients were divided into 2 groups (high and low) by the median value of the serum anti-PD-1 autoantibody (anti-PD-1 AAb) levels. (B) The Kaplan-Meier curve for the PFS among all patients divided into 2 groups according to the serum anti-PD-1 AAb levels (high and low). PFS was not affected by the serum anti-PD-1 AAb levels in all patients. The *P* value was determined by the Wilcoxon test. (C) The Kaplan-Meier curve for OS among all patients divided into 2 groups according to the serum anti-PD-1 AAb levels (high and low). The OS among patients who had lower serum anti-PD-1 AAb levels showed better OS rates, but the difference was not statistically significant. The *P* value was determined by the Wilcoxon test.

Next, we examined the treatment response, PFS, and OS in all patients. In this case, contrary to our hypothesis, serum anti-PD-1 AAb levels did not affect the outcome of Atezo/Bev therapy (Figure 1). Although the OS rates were better among patients with lower serum anti-PD-1 AAb levels, the difference was not statistically significant (Figure 1C).

**Figure 2:**
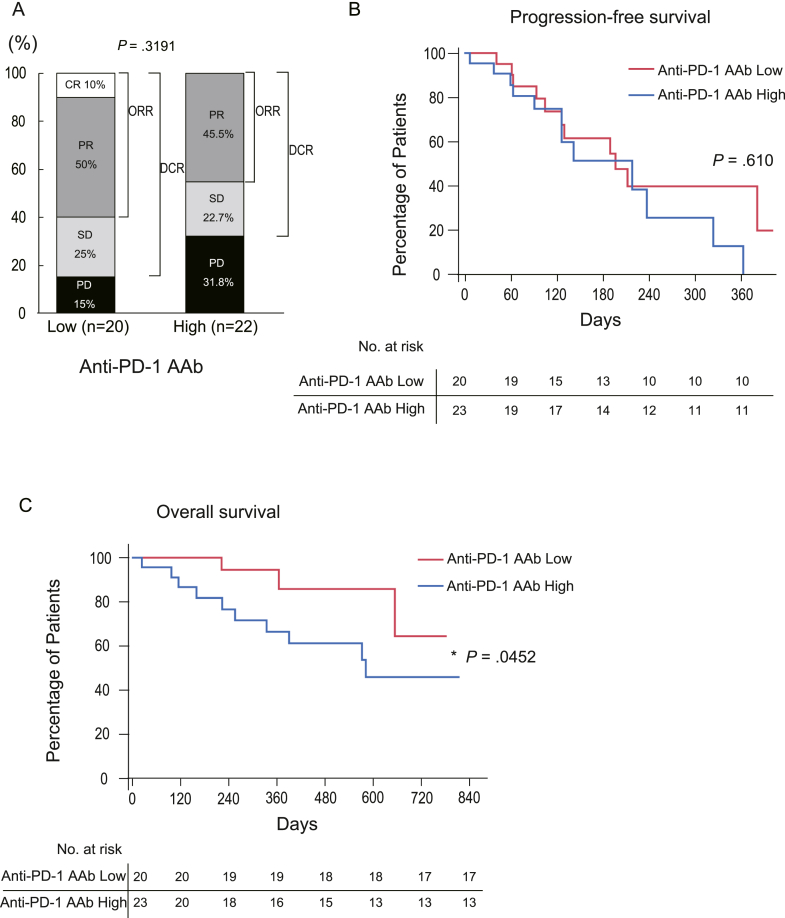
Patients with lower anti-PD-1 autoantibody levels showed better overall survival when Atezo/Bev was administered as their first-line therapy. (A) The bar chart showing the best response to Atezo/Bev therapy as the first-line treatment, determined by the radiological assessment, among the patients in the 2 groups (high and low) by the median value of the serum anti-PD-1 autoantibody (anti-PD-1 AAb) levels. (B) The Kaplan-Meier curve for the PFS among the patients who used Atezo/Bev as the first-line therapy, when divided into 2 groups according to the serum anti-PD-1 AAb levels (high and low). PFS was not statistically affected by the serum anti-PD-1 AAb levels among those patients. (C) The Kaplan-Meier curve for the OS among the patients who received Atezo/Bev as the first line therapy, when divided into 2 groups according to the serum anti-PD-1 AAb levels (high and low). The OS among those patients who had lower serum anti-PD-1 AAb levels showed significantly better OS rates. The *P* value was determined by the Wilcoxon test.

We subsequently examined patients who received Atezo/Bev as first-line chemotherapy. In this subgroup, the ORR and DCR were 60.0% and 85.0%, respectively, in patients with lower anti-PD-1 AAb levels (CR/PR/SD/PD: 2/10/5/3) and 45.5% and 68.2%, respectively, in patients with higher anti-PD-1 AAb levels (CR/PR/SD/PD/not evaluable: 0/10/5/7/1) (Figure 2A). Surprisingly, at the first radiologic response evaluation, 31.8% of patients with higher serum anti-PD-1 AAb levels and 15.0% of patients with lower anti-PD-1 AAb levels showed PD. Although there were no significant differences in the PFS between the 2 groups (Figure 2B), patients with lower serum anti-PD-1 AAb levels had significantly better OS rates than those with higher serum anti-PD-1 AAb levels (Figure 2C). These results suggest that high serum anti-PD-1 AAb levels may have deleterious effects on Atezo/Bev therapy only regarding the OS rate among patients who used it as first-line chemotherapy.

The anti-PD-1 AAb levels were examined in 4 CR and 5 PD cases by comparing the levels before the treatment and at the first evaluation time (Figure A2) to determine whether they changed during the Atezo/Bev therapy. The titer did not change significantly in either group.

### Factors Related to the OS Rate Among the Patients Using Atezo/Bev as the First-Line Therapy

Univariate Cox proportional hazards analyses showed that higher anti-PD-1 AAb levels and lower albumin levels were significantly associated with a poor OS rate in patients who received Atezo/Bev as their first-line therapy (Table 5). Because several studies have shown that NLR is associated with the prognosis of HCC and the PFS and OS of patients who received Atezo/Bev,^13^ we performed a multivariate analysis by adding NLR to both factors. Multivariate Cox proportional hazards analyses showed that the higher serum anti-PD-1 AAb levels (odds ratio [OR] = 7.8, 95% confidence interval [CI] = 1.5‒39, *P* = .013), the higher NLR levels (OR = 7.1, 95% CI = 1.6‒31, *P* = .009), and the lower albumin levels (OR = 14.2, 95% CI = 2.38‒84.9, *P* = .003) were independent risk factors for the poorer prognosis in patients with HCC who underwent Atezo/Bev therapy as the first-line regimen (Table 5). These results suggest that, similar to the NLR levels, serum anti-PD-1 AAb levels are also crucial factors for the prediction of the effectiveness of Atezo/Bev therapy when used as the first-line treatment.

**Table 5:** Cox Proportional Hazards Analyses Determining the Factors Related With the Overall Survival Rate Among Patients Who Received Atezo/Bev as Their First-Line Therapy

Characteristic	Univariate analysis (Wilcoxon)	Multivariate analysis (Cox)		
	*P* value	Hazard ratio	95% CI	*P* value
Age				
≥73	.734			
Sex				
Male/female	.216			
ECOG performance status				
>0	.552			
Etiology of HCC				
NASH/others	.265			
Child-Pugh score				
>5	.075			
PLT				
≥16.8	.526			
NLR				
≥2.7	.303	7.10	1.61–31.2	.009^a^
T-BiL				
≥0.79	.273			
Albumin				
<3.8	.003^a^	14.2	2.38–84.94	.003^a^
Cr				
≥0.74	.699			
Ferritin				
≥169	.254			
PT-INR				
≥1.06	.909			
AFP				
≥114	.383			
DCP				
≥350	.119			
Anti-PD-1 autoantibody				
≥0.57	.045^a^	7.82	1.54–39.6	.013^a^
Distant metastasis				
Present/absent	.306			
Vascular invasion				
Present/absent	.665			
BCLC stage				
A, B/C	.256			
Prior local therapy				
Present/absent	.275			
Protocols for the second line and subsequent therapies				
Present/absent	.198			

AFP, alfa-fetoprotein; Anti-PD-1, autoantibody, anti-programmed cell death-1, autoantibody; BCLC, Barcelona Clinic Liver Cancer; Cr, creatinine; DCP, des-γ-carboxy prothrombin; ECOG, Eastern Cooperative Oncology Group; HCC, hepatocellular carcinoma; NASH, nonalcoholic steatohepatitis; NLR, neutrophil lymphocyte ratio; PLT, platelets; PT-INR, prothrombin time-international normalized ratio; T-BIL, total bilirubin.

a *P* < .05.

## Discussion

In the present study, we determined the presence of anti-PD-1 autoantibodies in the sera of patients with advanced HCC. Patients with higher autoantibody levels had poorer OS rates with Atezo/Bev therapy than those with patients with the lower autoantibody levels when the therapy was used as the first-line regimen.

Abnormal autoantibodies (AAbs) may emerge in patients with cancers such as breast, lung, gastrointestinal, ovarian, and prostate.^21^ AAbs have advantages over other serum proteins as potential cancer biomarkers for their stability, high specificity, and ease of purification from serum. Therefore, AAb has been studied for the early detection of cancer,^22^ disease prognosis,^23^ and prediction of treatment response and side effects.^24^ For example, we have reported that AAb against cellular ribosomal protein L29 could be detected in the serum of patients with pancreatic cancer.^25^ In patients with unresectable pancreatic cancer, patients with higher anti-ribosomal protein L29 AAb levels showed better survival than those with lower levels, suggesting that the autoantibodies in cancer patients may be useful as a biomarker for predicting prognoses.

Anti-PD1 AAb has also been detected in patients with several types of cancers.^17^^,^^18^ However, its prognostic utility has not been reported, except in the cases of non-small cell lung cancer with a very limited number of patients examined.^18^ Similar to the study, we here determined that cases with higher anti-PD-1 AAb titers had worse OS rates, which appears contrary to the fact that externally administered anti-PD-1 agents generally improve prognoses. This may be because the external administration of anti-PD-1 antibodies may not be effective in patients with cancers that have progressed to advanced stages under the continuous presence of anti-PD-1 AAb, because these autoantibodies may antagonize the external anti-PD-L1 agents or downregulate the PD-1-associated signaling pathway.

One intriguing result of this study is that although anti-PD-1 AAb was predictive of OS, it was not predictive of PFS among patients who received Atezo/Bev therapy as the first-line regimen. A previous report on non-small cell lung cancer also showed similar results for PFS.^18^ The results showed that the median OS and PFS were similar to those of previous reports on Atezo/Bev therapy for HCC^7^; PFS and OS were not correlated with each other, which was also consistent with the results from ICI trials.^2^^,^^26^, ^27^, ^28^ In addition, the predictive power of the anti-PD-1 AAb titer in the serum regarding PFS and OS was not correlated. This may be because, in general, some ICI responders show prolonged responses to ICI therapy, even among patients with PD, resulting in prolonged OS. However, because they do not comprise the majority of treated patients, they do not contribute to prolonged PFS. Moreover, our cohort included patients who underwent conversion therapy, which resulted in favorable outcomes. However, in these patients, the PFS was terminated when the disease transiently progressed. Although the precise mechanisms for the discrepancy between OS and PFS remain to be elucidated, serum anti-PD-1 AAb level may be an effective marker for defining patients who can expect long-term survival, which is difficult to speculate from the PFS results.

Another intriguing point is that statistically significant differences in the OS rate were observed only among patients who received Atezo/Bev as the first-line therapy. Although not statistically significant, this tendency was also observed in all patients, irrespective of whether Atezo/Bev was used as the first-line therapy, suggesting that the significance of the utility of anti-PD-1 AAb as a biomarker for the prediction of the OS rate may be applicable to all patients. Although the anti-PD1 AAb level might be useful in defining the first-line protocol, whether the AAb levels can differentiate other agents’ effectiveness is unknown. Additional studies are required to observe a greater number of patients or to redefine the threshold of anti-PD-1 AAb levels.

However, the mechanism by which AAb is produced remains unclear. In autoimmune diseases such as systemic lupus erythematosus, anti-nuclear antibody positivity reflects disease activity.^29^ High anti-nuclear antibody titer has been shown to be correlated with strong immune responses.^30^ In ovarian cancer patients, the tumor and surrounding microenvironmental inflammatory responses have been shown to be correlated with autoantibody titers.^31^

In the present study, patients with higher anti-PD-1 AAb levels exhibited male predominance and higher serum ferritin levels, compared to those with low anti-PD-1 AAb levels in the first-line treatment group. Ferritin levels have been known to be higher in males.^32^ High ferritin levels in chronic liver diseases are due to hepatic necro-inflammation.^33^ The high anti-PD-1-AAb status may be associated with local liver damage and inflammatory responses. The patients in this study with high anti-PD-1 AAb relative values may have inflammatory changes in tumors and their microenvironment, resulting in AAb production. In the whole patient group, patients with higher anti-PD-1 AAb levels additionally exhibited low NLR and low frequency of prior local treatment experience. While the precise mechanisms of these phenomena are not clear, both resection and RFA have been reported to affect immune microenvironment. Especially if the RFA treatment was incompletely conducted, heat stress induces cancer stemness or production of immune suppressive chemokines such as CCL2, resulting in tumor progression and reduction in the effectiveness of immune checkpoint inhibitors.^34^ Such microenvironmental changes induced by locoregional therapies might have affected the anti-PD-1 AAb levels. Examining the histological stages of inflammation and the corresponding PD-1/PD-L1 expression levels in the tumor microenvironment may be required in the future.

This study had several limitations. First, the number of patients included in the study was limited. The correlation between AAb titers and clinical parameters should be determined in a larger cohort. Second, although this is a multicenter study, there was no racial diversity. Third, the observation period was too short to evaluate the overall survival completely. Finally, functional studies focusing on AAbs and their interactions with antibody drugs need to be conducted to clarify the biological significance of such AAbs.

## Conclusion

This study reported for the first time that higher serum anti-PD-1 AAb levels in patients with HCC correlated with a poorer prognosis, which may serve as a potential biomarker for OS when using anti-PD-L1 therapy as the first-line treatment against HCC. However, because little is known about the association between self-active anti-PD-1 AAb and clinical parameters and their biological roles in immunotherapy, the biology of AAb requires further clarification.

## Supplementary Data

Figure A1 Treatment responses among all patients or among the subgroup patients who received Atezo/Bev as the first line therapy. (A) The bar chart showing the best response to Atezo/Bev therapy determined by the radiological assessment among 62 patients (one patient could not be evaluated). (B) The Kaplan–Meier curve for overall survival (OS) and progression-free survival (PFS) among all 63 patients who received Atezo/Bev therapy. (C) The bar chart showing the best response to Atezo/Bev therapy determined by the radiological assessment in 42 patients who received Atezo/Bev as the first line therapy (one of the 43 patients could not be evaluated). (D) The Kaplan–Meier curve for OS and PFS among the 43 patients who received Atezo/Bev as the first-line therapy. PD, progressing disease; SD, stable disease; PR, partial response; CR, complete response.

Figure A2 Follow-up data of the anti-PD1-autoantibody levels. Four CR and 5 PD cases' follow-up serum samples were obtained from the first-line therapy group at the first evaluation. These samples examined changes in anti-PD-1 AAb titers between before the treatment and at the first evaluation. The *P*-value was determined by the Wilcoxon signed rank test.
