# Supplementary figures and images for: Anti-PD-1 Autoantibody Predicts Survival of Patients With Hepatocellular Carcinoma Receiving Atezolizumab/Bevacizumab
Source: Gastro Hep Adv. 2024 Aug 2;3(8):1138–47. doi: 10.1016/j.gastha.2024.07.018 (PMC11570718; doi:10.1016/j.gastha.2024.07.018)

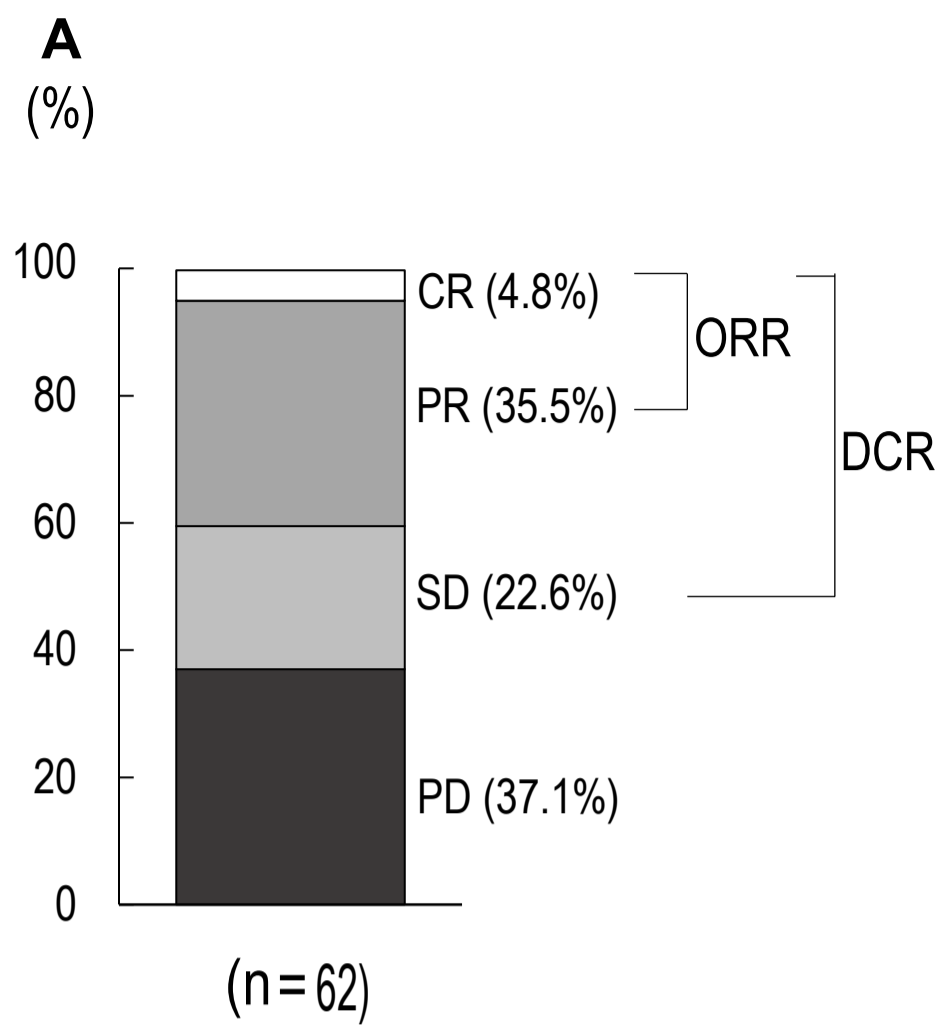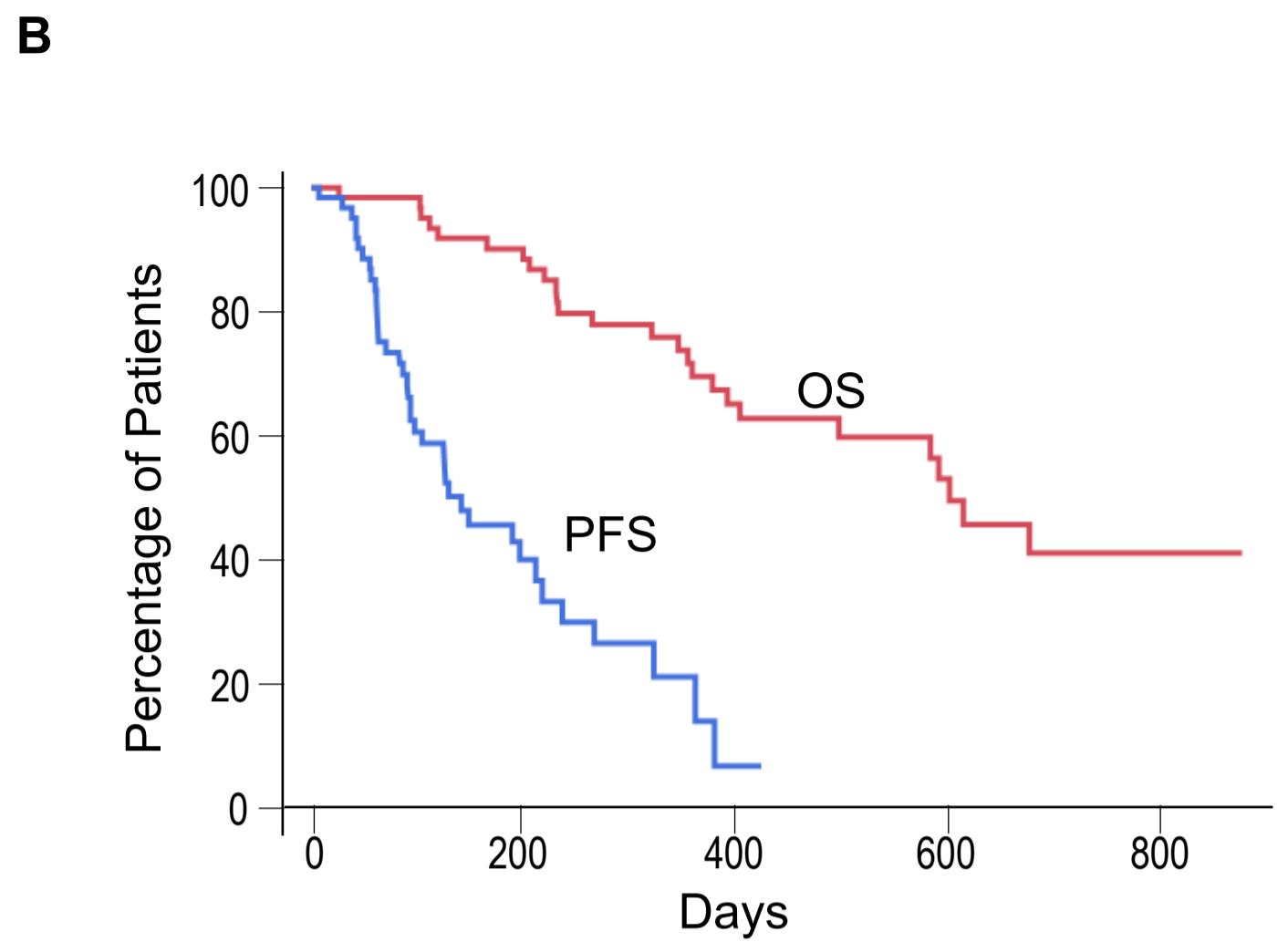

No. at risk

|     |    |    |    |    |    |    |    |    |    |
|-----|----|----|----|----|----|----|----|----|----|
| PFS | 63 | 39 | 30 | 26 | 23 |    |    |    |    |
| OS  | 63 | 62 | 56 | 50 | 44 | 42 | 39 | 37 | 37 |

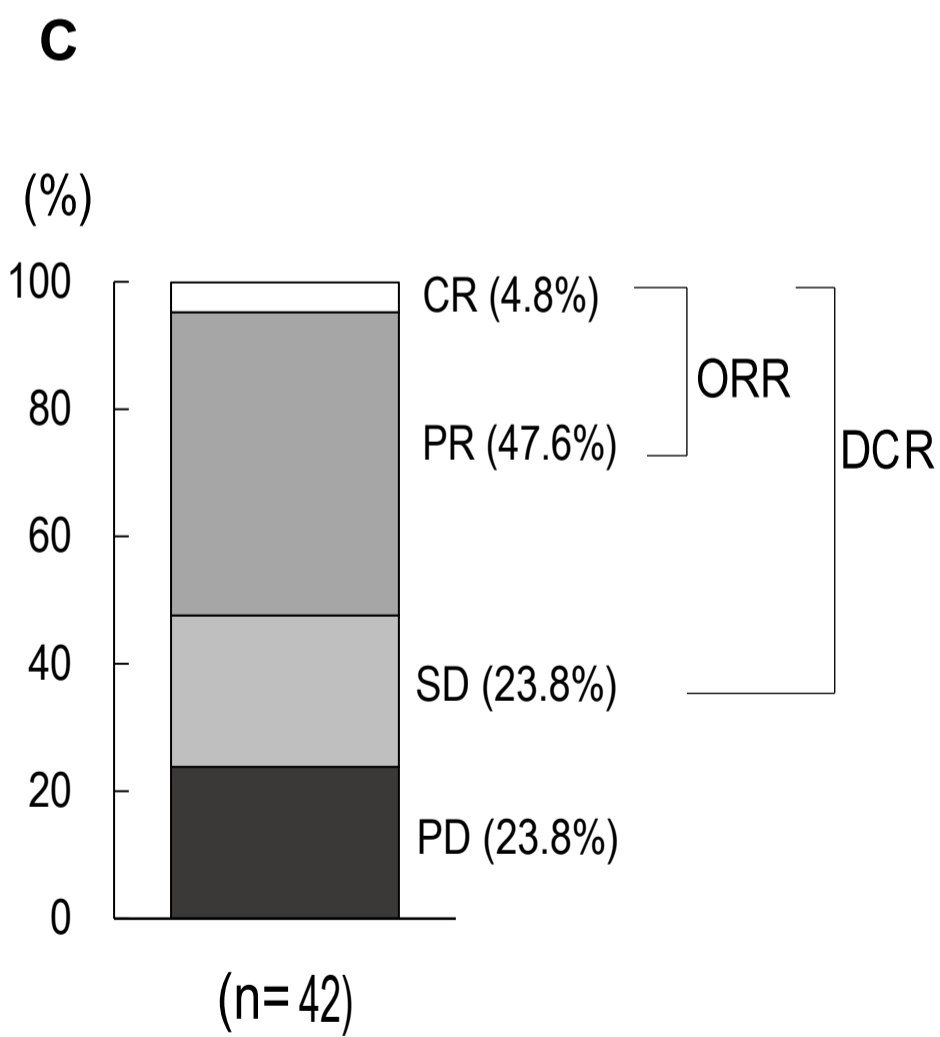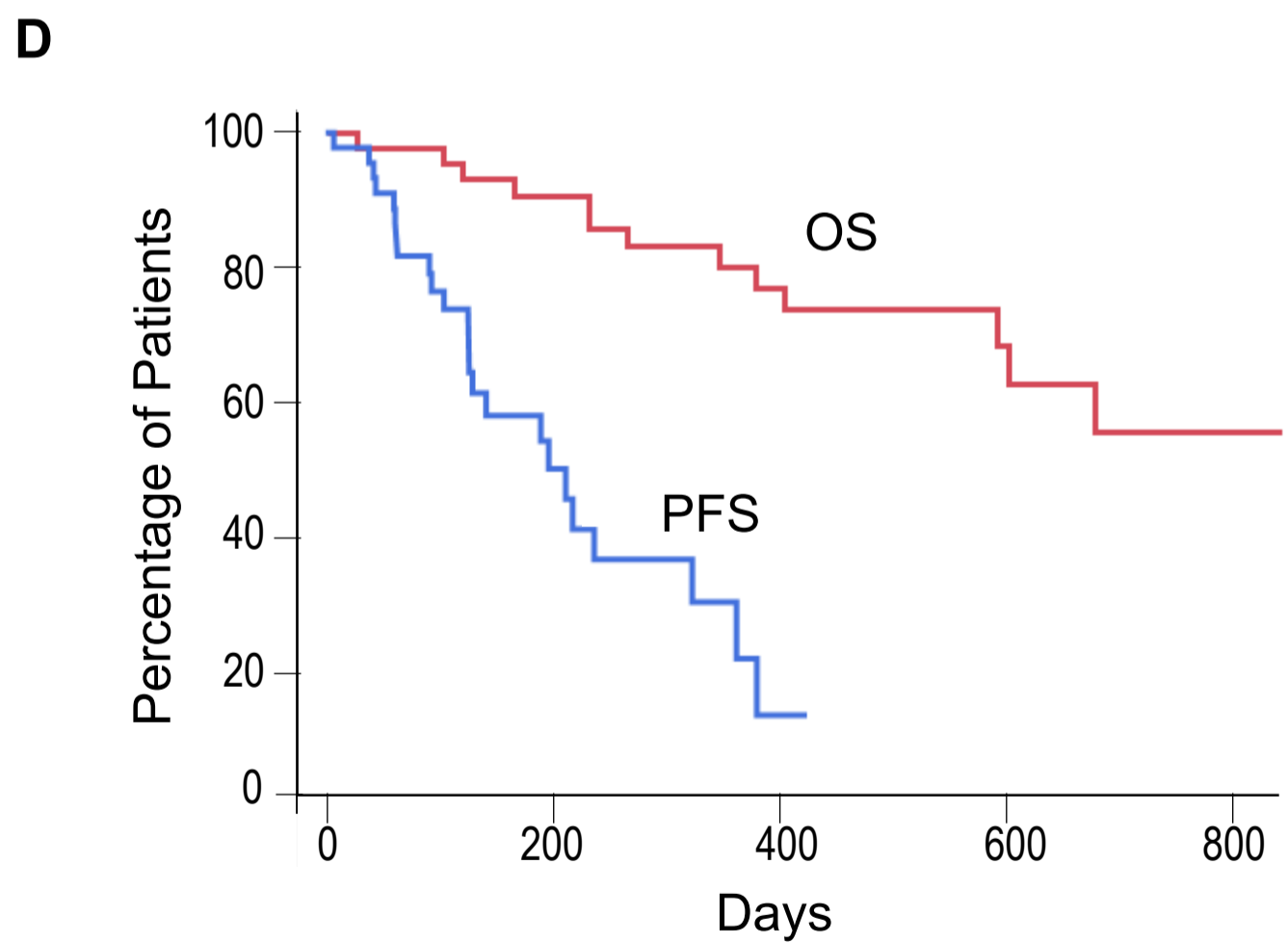

No. at risk

|     |    |    |    |    |    |    |    |    |    |
|-----|----|----|----|----|----|----|----|----|----|
| PFS | 43 | 33 | 25 | 22 | 19 |    |    |    |    |
| OS  | 43 | 42 | 39 | 36 | 34 | 33 | 32 | 30 | 30 |

Supplement: Figure A1 — Treatment responses among all patients or among the subgroup patients who received Atezo/Bev as the first line therapy. (A) The bar chart showing the best response to Atezo/Bev therapy determined by the radiological assessment among 62 patients (one patient could not be evaluated). (B) The Kaplan–Meier curve for overall survival (OS) and progression-free survival (PFS) among all 63 patients who received Atezo/Bev therapy. (C) The bar chart showing the best response to Atezo/Bev therapy determined by the radiological assessment in 42 patients who received Atezo/Bev as the first line therapy (one of the 43 patients could not be evaluated). (D) The Kaplan–Meier curve for OS and PFS among the 43 patients who received Atezo/Bev as the first-line therapy. PD, progressing disease; SD, stable disease; PR, partial response; CR, complete response. [file mmc1.pdf]

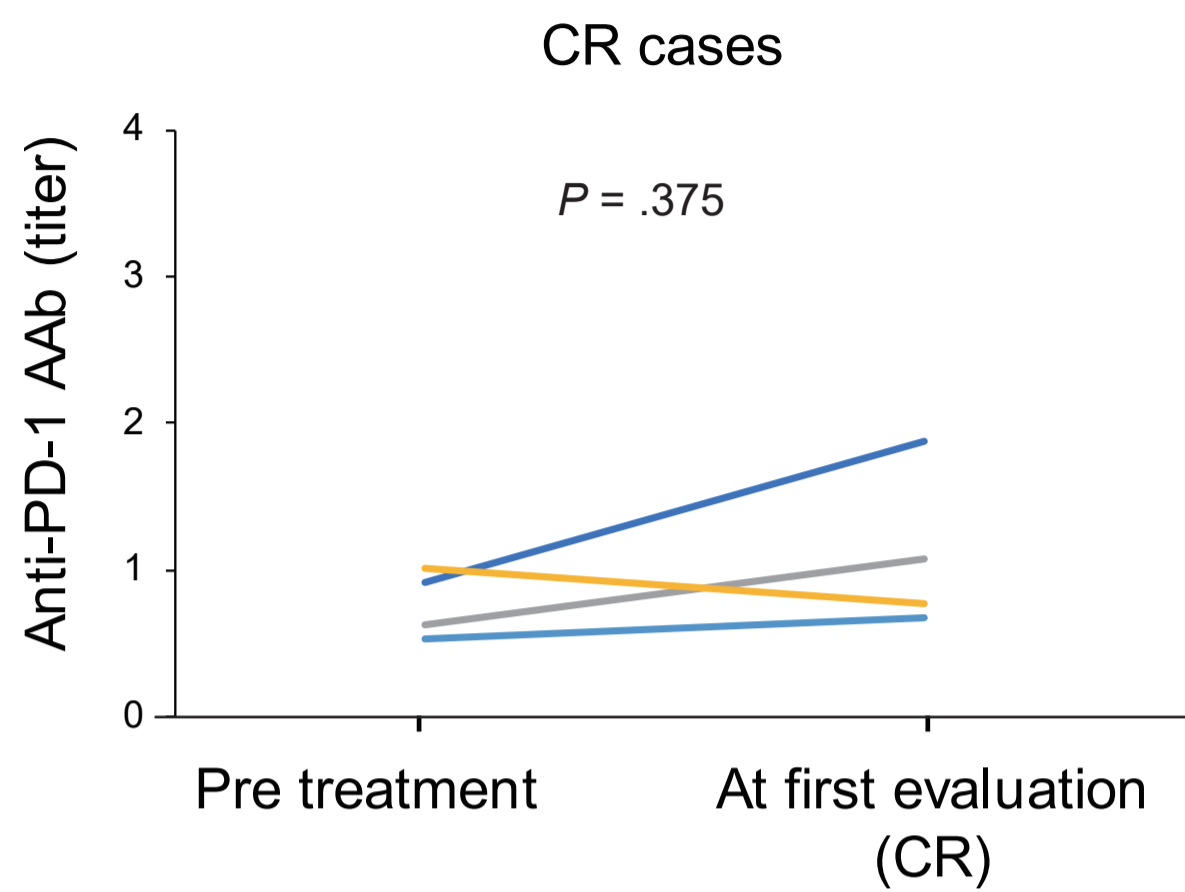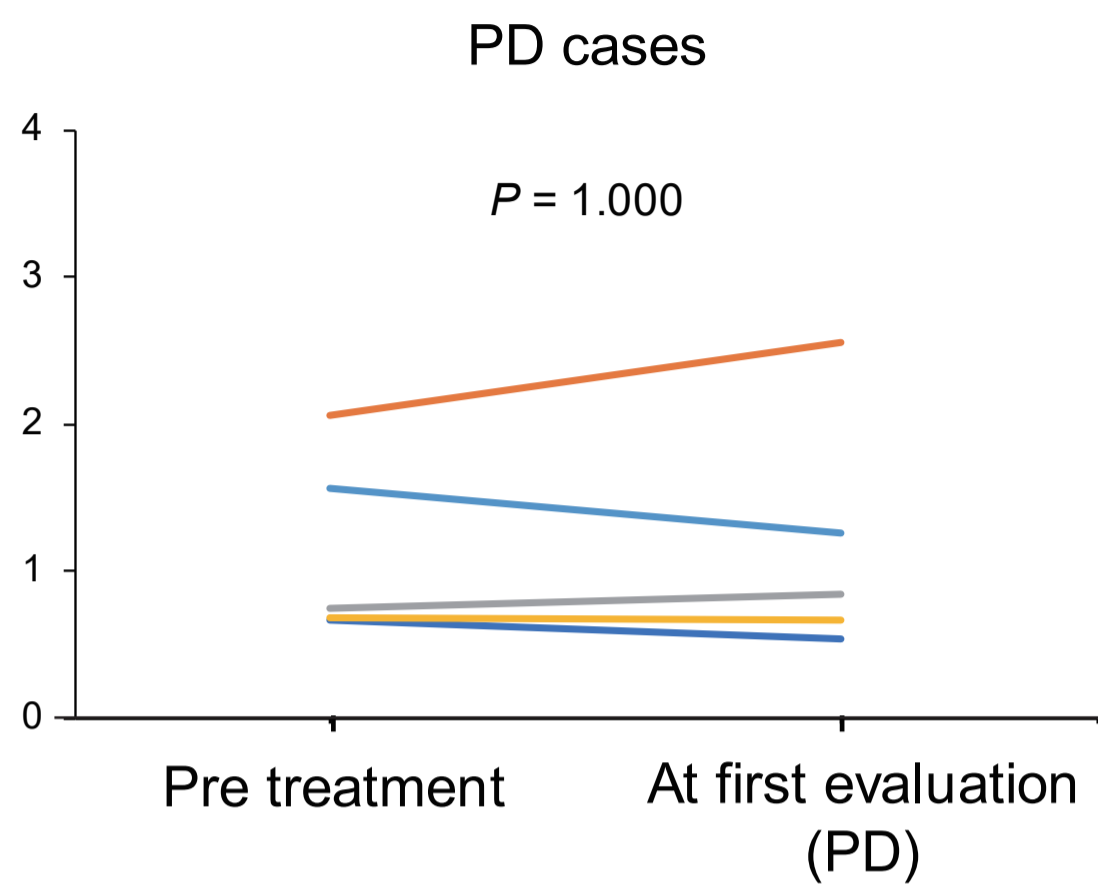

Supplement: Figure A2 — Follow-up data of the anti-PD1-autoantibody levels. Four CR and 5 PD cases' follow-up serum samples were obtained from the first-line therapy group at the first evaluation. These samples examined changes in anti-PD-1 AAb titers between before the treatment and at the first evaluation. The P-value was determined by the Wilcoxon signed rank test. [file mmc2.pdf]
